# Development and Validation of a Spanish Version of the Grit-S Scale

**DOI:** 10.3389/fpsyg.2018.00096

**Published:** 2018-02-07

**Authors:** Jose L. Arco-Tirado, Francisco D. Fernández-Martín, Rick H. Hoyle

**Affiliations:** ^1^Department of Development and Educational Psychology, University of Granada, Granada, Spain; ^2^Department of Psychology and Neuroscience, Duke University, Durham, NC, United States

**Keywords:** grit, non-cognitive skills, goal pursuit, scale validation, passion and perseverance

## Abstract

This paper describes the development and initial validation of a Spanish version of the Short Grit (Grit-S) Scale. The Grit-S Scale was adapted and translated into Spanish using the Translation, Review, Adjudication, Pre-testing, and Documentation model and responses to a preliminary set of items from a large sample of university students (*N* = 1,129). The resultant measure was validated using data from a large stratified random sample of young adults (*N* = 1,826). Initial validation involved evaluating the internal consistency of the adapted scale and its subscales and comparing the factor structure of the adapted version to that of the original scale. The results were comparable to results from similar analyses of the English version of the scale. Although the internal consistency of the subscales was low, the internal consistency of the full scale was well-within the acceptable range. A two-factor model offered an acceptable account of the data; however, when a single correlated error involving two highly similar items was included, a single factor model fit the data very well. The results support the use of overall scores from the Spanish Grit-S Scale in future research.

## Introduction

People vary in their typical response to adversity or failure when pursuing important long-term goals. Whereas some people are steadfast in their pursuit of such goals, sometimes over years, or decades, other people abandon pursuit in the face of significant challenges to the attainment of such goals. This variability is captured by the grit construct, defined as trait-level passion for and perseverance in pursuing long-term goals (Duckworth et al., 2007).

Within the Big Five taxonomy of personality traits, grit shares content with conscientiousness domain (Rimfeld et al., 2016) as reflected in its conceptual relatedness to several of the conscientiousness facets—orderliness, dependability, self-control, and industriousness (Duckworth and Eskreis-Winkler, 2015). Indeed, grit is view by some personality theorists as falling with the broad family of conscientiousness constructs (Roberts et al., 2014). Yet, despite its conceptual similarity to facets of conscientiousness, none of the conscientiousness facets fully captures the combination of passion and perseverance that characterizes grit. In particular, the sustained interest in important long-term goals, a core feature of grit, is not evident in conscientiousness or its facets (Sartori et al., 2017). Thus, although the conceptual overlap between the broad conscientiousness domain and the relative narrow grit trait is substantial, conceptually speaking, grit includes a specific and unique focus on the pursuit of long-term, higher-order goals.

The predictive validity of grit is evident in its prospective association with consequential outcomes that involve persistence in the pursuit of important goals. For example, controlling for cognitive ability, grit predicts educational attainment and fewer career changes in adults and grade point average, hours spent watching television (inversely), and retention in adolescents (Duckworth and Quinn, 2009). People higher in grit are more likely to persist on boring tasks and less likely to drop out of college (Yeager et al., 2014). Among new teachers, grit is associated with higher efficacy and greater retention (Duckworth et al., 2009). People higher in grit show greater work engagement (Eskreis-Winkler et al., 2014b), a reduced likelihood of changing professions or jobs (Duckworth et al., 2007; Eskreis-Winkler et al., 2014a), more stable marriages (Eskreis-Winkler et al., 2014a), less counterproductive behaviors (Ceschi et al., 2016), and higher scores on characteristics positively associated with life expectancy (Kim and Lee, 2015). Although the number of studies showing incremental prediction by grit is substantial, the contribution of grit is modest and may attributable primarily to the perseverance feature of the construct (Credé et al., 2017). Of particular concern is the contribution of grit beyond conscientiousness, though, as noted, conscientiousness is a very broad personality construct that encompasses some features of grit as well as other lower-order variables. Grit is relevant primarily for the pursuit of challenging, higher-order goals and, as would be expected, outperforms conscientiousness in the prediction of behaviors that contribute to progress toward achieving such goals (e.g., Tedesqui and Young, 2018).

Individual differences in grit are typically captured using self-report questionnaires. The original Grit Scale comprised 12 items, with half reflecting consistency of interest (i.e., passion) and half reflecting perseverance of effort (i.e., persistence; Duckworth et al., 2007). The focus of the current work is an abbreviated, eight-item version of the scale [Short Grit (Grit-S)], which has better psychometric properties than the longer version, including superior internal consistency, test-retest stability, and convergent and discriminant validity (Duckworth and Quinn, 2009). Factor analyses of both forms of the measure have produced support for the intended two-factor structure, with a moderate correlation between the two dimensions (Duckworth and Eskreis-Winkler, 2015). Although the passion and persistence items often are scored separately, the authors intended for the scale to be used to measure a single compound trait (e.g., Duckworth and Quinn, 2009).

Given the relevance of grit for consequential outcomes in the educational, personal, and professional domains, it is not surprising that the original and short grit scales are widely used across research topics and disciplines. Early work with the construct almost exclusively involved English-speaking respondents; however, recent years have witnessed multiple adaptations of one or both of the measures, including versions in German (Fleckenstein et al., 2014), Korean (Kim and Lee, 2015), Japanese (Nishikawa et al., 2015), Turkish (Akin et al., 2011), and Russian (Tyumeneva et al., 2014). Conspicuously absent is an adaptation of the instrument for use in research involving Spanish-speaking populations.

The objective of the current study was to adapt the Grit-S scale for Spanish-speaking respondents and document its psychometric properties. We expected that a Spanish language Grit-S Scale would evidence internal consistency values similar to those reported for the original Grit-S Scale (Duckworth and Quinn, 2009). Moreover, we expected factor analyses of the adapted items to show that responses could be attributed to two correlated factors, with factor loadings resembling those reported by Duckworth and Quinn (2009) for the English-language Grit-S Scale (cf. Muenks et al., 2017).

## Methods

The Grit-S Scale items were adapted to be used in a large-scale investigation of the cultural pathways to economic self-sufficiency and entrepreneurship and the role that family values play in youth unemployment in 10 European countries. The adapted items were included in the survey administered to young adults in Spain.

### Participants

As part of the adaptation procedure, detailed below, a sample of 1,129 university students responded to an initial translation of the scale (Fernández-Martín et al., 2017). The respondents' average age was 21 years old (*SD* = 1.7) and ranged from 18 to 52; 75% were women. Because some of the items evaluated at this stage were significantly altered to produce the final version of the scale, responses from this sample were not used in the psychometric analyses.

For the primary analyses, a proportional stratified random sampling technique was used with regions (i.e., Nomenclature of Units for Territorial Statistics-2), employment status, age, and sex serving as strata. The resultant sample of 1,826 adults ranged from 18 to 35 years old, with an average age of 27.56 years (*SD* = 5.00), and a distribution by sex comprising 893 men (48.90%) and 933 women (51.10%). Almost 70% of the respondents had relatively high levels of education and fewer than a half were employed or self-employed.

### Adaptation and administration

The procedure to adapt the Grit-S scale followed the recommendations established by specialized literature (Carretero-Dios and Pérez, 2007; Sartori and Pasini, 2007; Muñiz et al., 2013). We followed the translation recommendations in the Cross-Cultural Survey Guidelines, adhering closely to the TRAPD (Translation, Review, Adjudication, Pretesting, and Documentation) team translation model (Survey Research Center, 2016). Our implementation of the model began with a parallel translation from English into Spanish by two bilingual translators with ample experience translating evaluation instruments. Next, a revision committee, composed of the translators and one of the researchers, who is bilingual and a specialist in the development of surveys, analyzed the linguistic, and cultural equivalence of the translations independently produced by the two translators with the original Grit-S Scale. The translated and original items were designated as either: (a) identical, if the meaning of the item and the observed changes were the same, (b) similar if there were changes in the meaning of any of the words but not on the item, or (c) different when there was changes in the meaning of the original item because of the cultural adaptation. Then, the discrepancies derived from the analysis of the linguistic equivalence between members of the revision committee were resolved with a second round revision of the items by comparing the original version of the Grit-S Scale with the translation and the revision of the items by the translators and the reviewer together. Finally, a pilot test was implemented with the Spanish version of the Grit-S scale with the sample of university students, whose responses allowed us to examine and to modify some issues related to the implementation of the adaptation process. In particular, due to several queries posed by participants about the concept “diligent” [“diligente”] in item 8, a note to clarify the meaning was added [i.e., Having or showing care and conscientiousness in one's work or duties (es decir, cuidadoso, activo y que ejecuta con celo y exactitud lo que está a su cargo)]. Furthermore, item number 2, “Los contratiempos no me desaniman” [Setbacks don't discourage me.], was rephrased because the double negative present in the pilot-tested item (and in the original item) in combination with the *Strongly disagree* response option indicative of high grit, caused confusion for some respondents as evidenced by a loading of only 0.19 on perseverance factor.

The resultant Spanish version of the Grit-S Scale comprises eight items, to which responses are indicated on four-point scales labeled 1 (*Strongly disagree*), 2 (*Somewhat disagree*), 3 (*Somewhat agree*), and 4 (*Strongly agree*). As with the original version of the scale (Duckworth and Quinn, 2009), the items arrayed in two sets corresponding to the core features of the grit construct, consistency of interest and perseverance of effort. The items are displayed in Table 1.

**Table 1:** Items of the Spanish and Original English Version of the Grit-S Scale arrayed by subscale.

**Consistencia del interés**	**Consistency of Interest**
1. Las ideas y proyectos nuevos a veces me distraen de ideas y proyectos anteriores.	1. New ideas and projects sometimes distract me from previous ones.
3. He estado obsesionado/a con alguna idea o proyecto durante un tiempo breve, pero después he perdido el interés.	3. I have been obsessed with a certain idea or project for a short time but later lost interest.
5. A menudo me pongo una meta pero después cambio a otra diferente.	5. I often set a goal but later choose to pursue a different one.
6. Tengo dificultades para mantener mi atención en proyectos que requieren más de unos meses en completarse.	6. I have difficulty maintaining my focus on projects that take more than a few months to complete.
**Perseverancia en el esfuerzo**	**Perseverance of Effort**
2. Los contratiempos me desaniman.	2. Setbacks don't discourage me.
4. Soy muy trabajador/a.	4. I am a hard worker.
7. Termino siempre todo lo que empiezo.	7. I finish whatever I begin.
8. Soy diligente (es decir, cuidadoso, activo y que ejecuta con celo y exactitud lo que está a su cargo)	8. I am diligent.

Once the adaptation was completed, a polling firm, in collaboration with the research team, used the Computer-Assisted Web Interviewing (CAWI) technique to recruit a stratified random sample of members of a panel. Prospective respondents were sent an online invitation and given 14 days to respond. The website through which CAWI was implemented was compatible with different browsers, operating systems, and screen resolutions, and featured a simple and attractive design with security and support mechanisms (Couper and Bosnjak, 2010; Haer and Meidert, 2013). The invitation to panel members provided information on the objectives of the research, the voluntary nature of their participation, and the confidentiality of their responses. Panel members who elected to participate were provided a respondent-specific link to access the Spanish version of the Grit-S Scale. Responses were collected from February to June of 2016.

### Data analysis

We evaluated the new Spanish version of the Grit-S Scale in two stages. We first computed coefficient alpha for the full scale and the two subscales. Values were interpreted against normative standards and in comparison to values generated in research using the original scale. We then examined the latent structure of the items using confirmatory factor analysis. The a priori model specified two correlated factors corresponding to the item sets identified in Table 1.

We estimated and tested the model using the maximum likelihood estimator as implemented in EQS version 6.3. Although the univariate distributions were approximately normal (see the two rightmost columns in Table 2), Mardia's coefficient indicated a modest departure from multivariate normality. Thus, we used robust estimation and scaled statistics as provided in EQS (Satorra and Bentler, 1988). We evaluated the fit of the model by using two widely-used and well-validated fit indices (Hu and Bentler, 1999). The comparative fit index (CFI; Bentler, 1990) indexes the proportionate improvement in fit of a specified model over a baseline model in which the observed variables are independent; values between 0.90 and 1.00 are expected for measurement models that fit the data well (Hu and Bentler, 1999). The root mean square error of approximation (RMSEA; Steiger and Lind, 1980) indexes degree of misspecification in a model per degree of freedom, with a value of zero indicating perfect fit to the data and values from zero to 0.10 indicating acceptable fit (Browne and Cudeck, 1993). We put 90% confidence intervals on the point estimate, expecting the upper limit to fall below 0.10 for a good-fitting model. As is typical, we report but do not interpret the chi-square approximation associated with individual models. These values are used for comparisons of nested models, for which they are appropriate (Steiger et al., 1985). After evaluating a priori models, we examined values of a model modification index in search of residual covariance unaccounted for by the best-fitting a priori model (Chou and Bentler, 1990).

**Table 2:** Correlations between Translated Grit-S Items and Item-Level Descriptive Statistics.

**Subscale/Item**	**Item 2**	**Item 4**	**Item 7**	**Item 8**	**Item 1**	**Item 3**	**Item 5**	***M***	***SD***	**Skewness**	**Kurtosis**
**PERSEVERANCE OF EFFORT**											
Item 2								2.289	0.826	0.34	−0.35
Item 4	0.046							3.519	0.655	−0.09	−0.66
Item 7	0.108	0.324						3.092	0.794	−0.45	−0.53
Item 8	0.098	0.388	0.315					3.423	0.638	−0.74	−0.14
**CONSISTENCY OF INTEREST**											
Item 1	0.310	0.187	0.299	0.213				2.544	0.860	0.08	−0.67
Item 3	0.233	0.105	0.196	0.130	0.382			2.437	0.905	0.04	−0.78
Item 5	0.272	0.157	0.266	0.210	0.477	0.428		2.625	0.863	−0.09	−0.66
Item 6	0.329	0.280	0.299	0.276	0.560	0.399	0.545	2.877	0.924	−0.38	−0.76

*All correlation coefficients differ from zero at p < 0.05*.

Following evaluation of omnibus fit, we examined parameter estimates, with a particular focus on factor loadings. As with alpha, we interpreted values of loadings with reference to normative standards and in comparison to loadings from similar analyses of the original Grit-S Scale.

## Results

Descriptive statistics and correlations between the items are presented in Table 2. The correlations between item 2 and the other items on the perseverance of effort subscale are quite low. Otherwise the correlations range in magnitude from modest to moderate.

Internal consistency estimates for the translated items appear in Table 3. In the top portion of the table are estimates for the current sample. Below these appear representative values of alpha for English-speaking samples who completed the original version of the Grit-S Scale. Looking first to the values for the full set of items, the value of alpha for the current sample is comparable to corresponding values for the comparison samples. Moving to the subscales, the value of alpha for the consistency of interest items is strong and higher than values for three of the four comparison samples. Alpha for the perseverance of effort items is relatively low both in absolute terms and compared to values for the comparison samples, reflecting the low correlations between item 2 and other items in the set (the value of alpha rises to 0.63 if item 2 is omitted). Given the brevity of the subscales, even the low value for the perseverance of effort items is acceptable (i.e., corrected item-total *r*s range from 0.36 to 0.63 except item 2 = 0.08; Nunnally and Bernstein, 1994). For the full set of items, all corrected item-total *r*s, including for item 2, are >0.30 and would reduce the value of alpha if deleted.

**Table 3:** Internal Consistency of the Original English and Spanish Versions of the Grit-S Scale.

**Grit-S version**	**Population**	***N***	**Grit-S**	**Consistency of Interest**	**Perseverance of Effort**
**Spanish**	Adults between 18 and 35 years old	1826	0.75	0.77	0.48
**Original**	West Point 2008	1218	0.73	0.73	0.60
	West Point 2010	1308	0.76	0.74	0.65
	2005 National Spelling Bee	175	0.80	0.76	0.65
	Ivy League Undergraduates	139	0.83	0.79	0.78

Results of the omnibus test of the two-factor model of the translated scale are provided in the top portion of Table 4. As with the evaluation of internal consistency, we provide comparison values for English-speaking samples that completed the original version of the scale. The value of CFI falls within the range of acceptable values. The value of RMSEA meets criterion as well, with a point estimates and upper confidence limit below 0.10. Values of these indices are consistent with values for prior analysis of the original version of the scale shown in the lower portion of the table. In terms of omnibus fit, the two-factor measurement model provides an acceptable account of the data.

**Table 4:** Fit information for the Two-Factor Model for the Spanish and Original Versions of the Grit-S Scale.

**Grit-S versions/Population**		***N***	**χ^2^**	***df***	**CFI**	**RMSEA**	**90% Confidence Limits**
**Spanish**	Adults between 18 and 35 years old	1826	325.52	19	0.92	0.084	0.076, 0.093
**Original**	West Point 2008	1218	106.36	19	0.95	0.061	0.050, 0.073
	West Point 2010	1308	135.51	19	0.95	0.068	0.058, 0.080
	2005 National Spelling Bee	175	71.57	19	0.86	0.101	0.077, 0.126
	Ivy League Undergraduates	139	43.63	19	0.93	0.097	0.059, 0.135
	Adults aged 25 and older	1554	188.52	19	0.96	0.076	0.066, 0.086

Factor loadings are presented in Table 5. Loadings for the two-factor model are presented in the middle two columns. The loadings exceed 0.50 for all items except item 2, which, as noted earlier, shares less in common with the remaining perseverance of effort items than would be expected. Nevertheless, the loading for item 2 was noticeably higher than the loading for the initial version of that item on the perseverance factor in the pilot study. The correlation between the two factors was 0.62, which is somewhat higher than values in the 0.40–0.50 range observed in analyses of the original scale (e.g., Duckworth et al., 2007; Von Culin et al., 2014) but not inconsistent with the assumption that the factors are constituents of a single construct.

**Table 5:** Factor Loadings for the Two- and One-Factor Models.

**Item**	**Consistency of Interest**	**Perseverance of Effort**	**Grit**
1	0.696		0.696
3	0.535		0.541
5	0.697		0.694
6	0.795		0.792
2		0.271	0.408
4		0.557	0.301
7		0.561	0.379
8		0.582	0.282

Although the fit of the two-factor model met our omnibus fit criteria, the values of the fit indices, especially RMSEA, suggested that the fit of the model could be improved. As noted earlier, we used a modification index, specifically the Lagrange Multiplier test (Bentler, 1986) of fixed parameters. We focused specifically on covariances between uniquenesses, which convey commonality between items unaccounted for by the two factors. Those tests revealed significant covariance between items 4 (Soy muy trabajador/a., I am a hard worker.) and 8 (Soy diligente, I am diligent.) not accounted for by the perseverance of effort factor and therefore evident in the covariance between their associated uniquenesses. Estimation of a model in which that parameter was freed produced substantially improved fit statistics, scaled χ^2^(18, N = 1, 826) = 235.36, CFI = 0.94, RMSEA = 0.075 (CLs = 0.065, 0.083), and a highly significant single degree-of-freedom scaled-chi-square difference (Satorra and Bentler, 2001) of 77.90. The loadings on the consistency of interest factor were unaffected by this modification, but the loadings on the perseverance of effort factor differed noticeably: 419, 0.348, 0.436, and 0.326, for items 2, 4, 7, and 8, respectively. The correlation between the item 4-item 8 uniquenesses was 0.40 and, surprisingly, the correlation between the two factors rose to 0.89.

Given the very high correlation between the two factors in a model with a single correlated uniqueness, we estimated a one-factor model that included the correlated uniqueness. That model, which is consistent with the conceptual model of grit as a single construct comprising passion *and* perseverance, fit the data exceptionally well, scaled χ^2^(19, N = 1, 826) = 233.21, CFI = 0.95, RMSEA = 0.071 (CLs = 0.062, 0.080). The nested model comparison of the one- and two-factor models indicated minimal loss in fit from adding the simplifying constraint of perfect correlation between the factors, Δscaled χ^2^(1) = 4.68, *p* = 0.03. The loadings for the one-factor model with a correlation between uniquenesses for items 4 and 8 (*r* = 0.42) are presented in the rightmost column of Table 5.

## Discussion

Our goal was to develop a version of the Grit-S Scale for use in research involving Spanish speaking populations. Although the results of internal consistency analyses and confirmatory factor analysis were mixed, they support the use of the scale as originally intended—to assess a single, compound construct. The internal consistency of the full scale is consistent with that of the original scale, and, when a single residual covariance is accounted for, the fit of a single-factor model is excellent. This new version of the measure will help fill a gap in the literature on grit by enabling the inclusion of the Grit-S Scale in studies involving Spanish-speaking populations.

Although the reliability estimates and model fit statistics were in the acceptable range, they were not strong. Importantly, they were consistent with, and occasionally exceeded, comparable values for the original version of the scale as well as translated versions. With respect to reliability, a key consideration is how the measure is to be used. The estimate of internal consistency we observed for the full scale is both comparable to the same estimate in multiple studies using the original version of the scale and within the acceptable range for brief measures. Importantly, all items contribute to the overall reliability of the measure, with acceptable values of corrected-item correlations and a reduction in coefficient alpha if any item is deleted. The overall value of alpha and value of the inter-item correlations are consistent with a measure characterized by bandwidth as opposed to fidelity (Briggs and Cheek, 1986). That bandwidth, to a large degree, is attributable to the decision to separately assess consistency of interest and perseverance of effort by the original scale developers (Duckworth et al., 2007). In addition, the perseverance of effort items sample a broader domain space than the consistency of interest items, addressing setbacks, finishing what one starts, and hard work. A consequential exception to this bandwidth characterization is the somewhat redundant pair of consistency of interest items that separately refer to diligence and hard work. As our follow-up exploratory analyses revealed, this redundancy is evident in covariance between the responses to these items that is not captured by the perseverance of effort factor. It will be important to examine this feature of the measurement model in independent samples across the languages into which the scale has been translated. If, as we suspect, this residual covariance is evident across populations and translations, the measure would be improved by replacing one of the items with a new item that eliminates the redundancy.

Although the original conceptual model and the results of our analyses support the use of the Grit-S to produce a single score, some researchers may wish to produce separate scores for passion and perseverance. Although our results support producing a score for passion, they do not support doing so for perseverance. In particular, and consistent with recent analyses of the original items (Muenks et al., 2017), one of the perseverance items shares little variance with the others as demonstrated by a low corrected item-total correlation in the internal consistency analyses and a low loading in the two-factor model. Importantly, that item performs satisfactorily when internal consistency is evaluated for the full set of items and the responses are fit to a single-factor model. These findings underscore our recommendation that the scale be used to produce a single score, thereby avoiding concerns for this item and better reflecting the full grit construct (e.g., Jachimowicz et al., 2017).

Our promising findings should be interpreted in light of the strengths and limitations of our adaptation and initial evaluation of the translated items. In the absence of prior cultural and linguistic adaptations in Spanish, we were unable to compare our version of the scale with others as recommended (López-Walle et al., 2011). Our sampling strategy has strengths and potential limitations. Prior studies like ours (e.g., Akin et al., 2011; Nishikawa et al., 2015), including the study in which the original Grit-S scale was evaluated (Duckworth and Quinn, 2009), did not draw random samples, perhaps limiting the representativeness or generalizability of the findings. On the other hand, the representativeness of our sample is unclear because of the digital literacy levels necessary to respond to the survey.

Our evaluation of Spanish-language version of the Grit-S Scale suggests that, when used to generate a single score, the scale is comparable to the original scale. As such, we would expect findings using the measure to contribute the broader literature on the role of grit in the pursuit of long-term, higher-order goals. Future research could build on our initial evaluation through an examination of other psychometric properties of the instrument like the test-retest reliability and the predictive validity. That work might also consider the contribution of grit and its constituents to success in long-term goal pursuits in Spanish-speaking populations controlling for psychological and educational variables, as has been done in English-speaking populations with the original version of the scale.

## Ethics statement

This study was carried out in accordance with the recommendations of the ethics committees of NetQuest and UPF with written informed consent from all subjects. All subjects gave written informed consent in accordance with the Declaration of Helsinki. The protocol was approved by the ethics committees of NetQuest and UPF.

## Author contributions

JA-T and FF-M were responsible for adapting and validating the scale that was used later in a larger research project; both authors participated actively in the process of translating the original English scale into Spanish as well as supervised the implementation process of the survey; both authors clean the resulting database and did the statistical analysis necessary to validate the English version with Spanish population, including the discussion section and the references part. RH supervised and replicated the data analysis process as well as the reporting process of the results particularly the proofreading of the paper in English.

### Conflict of interest statement

The authors declare that the research was conducted in the absence of any commercial or financial relationships that could be construed as a potential conflict of interest.
